# Effect of photobiomodulation with isolated or combined use of red and infrared lasers on repair of sites grafted with deproteinized bovine bone

**DOI:** 10.1590/0103-644020256529

**Published:** 2025-12-08

**Authors:** Lucas de Sousa Goulart Pereira, Priscilla Barbosa Ferreira Soares, Isadora Aparecida Ribeiro Reis, Julia Raulino Lima, Caio Fossalussa Silva, Guilherme José Pimentel Lopes de Oliveira

**Affiliations:** 1 Department of Periodontology, School of Dentistry, Universidade Federal de Uberlândia- UFU, Uberlândia, MG, Brazil

**Keywords:** bone repair, bone substitute, adjunctive treatments, photobiomodulation

## Abstract

The current study aimed to evaluate different PBMT protocols using red and infrared lasers, both individually and in combination, for bone regeneration in sites grafted with deproteinized bovine bone (DBB). Forty-eight rats were evaluated in two experimental periods (30 and 90 days - n=6). A Teflon capsule was placed bilaterally in the jaw ramus of each animal and filled with DBB. The groups were divided according to the type of treatment applied to the grafted area: CTR: No adjunctive treatment; IRL: PBMT with an infrared laser; RL: PBMT with a red laser; IRL/RL: PBMT with an infrared and a red laser. The following analyses were performed: 1) Microtomography to evaluate the volume and microstructure of the grafted area; and 2) Histomorphometry to evaluate the composition of the repaired tissue in the grafted area. The IRL/RL group presented greater trabecular thickness than the CTR group at 90 days (p<0.05), while the CTR group presented a greater number of trabeculae than the IRL group at 90 days (p <0.05). Regarding histomorphometry analysis, the IRL/RL group presented a greater amount of bone at 90 days than all other groups (p<0.05) and a lower amount of soft tissue than the CTR and RL groups at 90 days (p<0.05). PBMT associated with the infrared and red lasers improves bone repair at DBB-grafted sites.

## Resumo

Esse estudo comparou o efeito da fotobiomodulação (PBMT) com luz vermelha e infravermelha de forma isolada ou associada sobre o reparo de áreas enxertadas com osso bovino desproteinizado (OBD). Foram utilizados 48 ratos que foram avaliados em dois períodos experimentais (30 e 90 dias - n=6). Foram instalados bilateralmente no ramo da mandíbula de cada animal uma cápsula de teflon que foi preenchida com OBD. Os grupos foram divididos de acordo com o tipo de tratamento aplicado a área enxertada: CTR: Sem tratamento adjunto; IRL: OBD associado a PBMT com luz infravermelha; RL: OBD associado a PBMT com luz vermelha; IRL/RL: OBD associado a PBMT com luz infravermelha e vermelha. Foram executadas as seguintes análises: 1) Microtomografia para avaliação do volume e da microestrutura da área enxertada 2) Histomorfometria para avaliação da composição do tecido reparado. Foi observado que o grupo IRL/RL apresentou maior espessura de trabéculas que o grupo CTR no período de 90 dias (p<0.05), enquanto que o grupo CTR apresentou maior número de trabéculas do que o grupo IRL no período de 90 dias (p<0.05). Em relação a análise histomorfométrica foi observado que o grupo IRL/RL apresentou maior quantidade de osso no período de 90 dias que todos os outros grupos (p<0.05) e menor quantidade de tecidos moles do que o grupo CTR e RL no período de 90 dias (p<0.05). A PBMT com laser infravermelho e vermelho melhora o reparo em áreas enxertadas com OBD.

**Figure:**
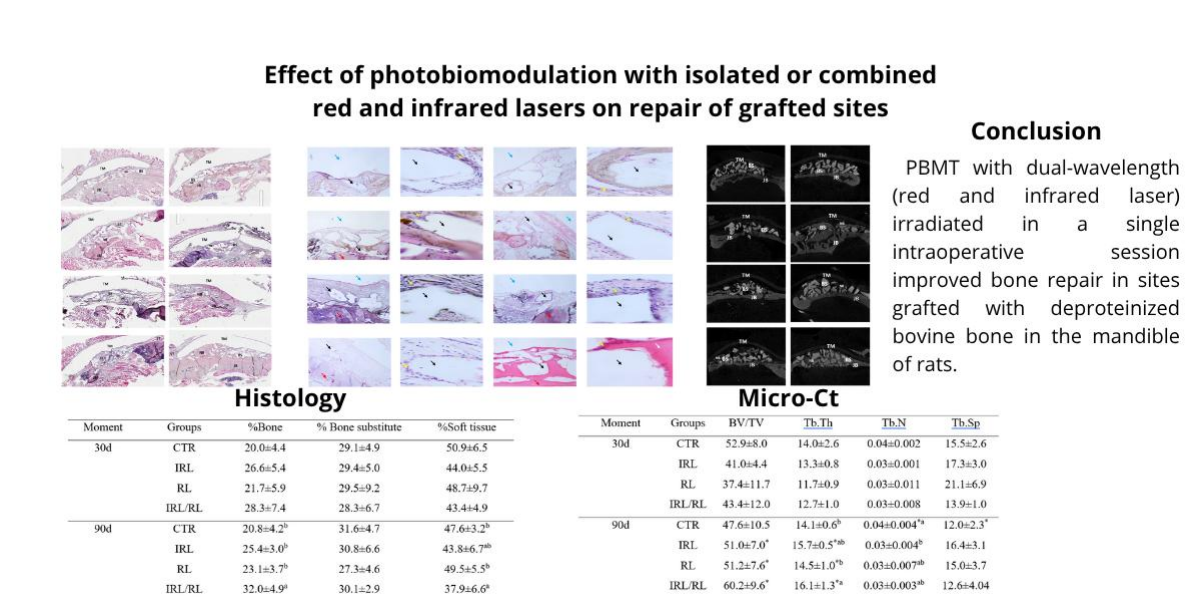

## Introduction

Bone defects in the alveolar ridge represent a significant challenge in dentistry, as they complicate or even prevent rehabilitation in edentulous areas ^1^ ^,^ ^2^. To restore bone volume and quality in these areas, bone substitutes have been recommended using the guided bone regeneration technique ^3^. Autogenous grafts are considered the gold standard due to their excellent biological properties ^4^; however, factors such as the patient's health condition, high postoperative morbidity, and limited availability limit the harvesting of autogenous bone tissue and its use in grafting techniques ^5^.

Among the various bone substitutes with osteoconductive capability, there is more scientific evidence supporting the use of deproteinized bovine bone (DBB) ^6^,^7^. However, despite favorable clinical results, DBB has a lower biological capacity compared to autogenous grafts ^8^. To enhance the biological capacity of osteoconductive bone substitutes, adjunctive treatments, such as growth factors ^9^, blood concentrates ^10^, and photobiomodulation, have been used in conjunction with various bone substitutes in grafted areas. Although adjunctive treatments using growth factors and blood concentrates yield good results, limitations such as the high cost of BMP-2 and the need for specific, as well as costly, training and equipment for PRF collection make photobiomodulation a favorable adjunctive treatment option in terms of the cost-benefit ratio ^10^, ^9^.

Photobiomodulation has shown promising findings in bone regeneration and fracture healing ^11^, ^12^. These results suggest that using photobiomodulation (PBMT) as an adjunct therapy improves bone regeneration in grafted areas. Studies with PBMT have been conducted using two different wavelengths. The infrared laser offers high tissue penetration, accelerating bone healing and osseointegration in areas grafted with osteoconductive biomaterials ^13^; however, the PBMT protocol requires 7 sessions, which can hinder patient adherence to the treatment. On the other hand, red light has shown promising results in bone regeneration in preclinical studies with immunosuppressed animals, using the laser in a single session during the trans-surgical period ^14^, ^15^. Further investigation is required into direct comparisons of red and infrared lasers, and into their combined use to accelerate bone regeneration in grafted areas, and into improving the irradiation protocol to reduce the number of sessions and, consequently, facilitate patient adherence to treatment.

In this context, the current study aimed to evaluate different PBMT protocols using red and infrared lasers, both individually and in combination, for bone regeneration in sites grafted with deproteinized bovine bone. The null hypothesis of this study is that there will be no difference in bone regeneration in areas grafted with deproteinized bovine bone using the different PBMT protocols.

## Materials and methods

This study was previously approved by the Animal Ethics Committee of the Federal University of Uberlândia (UFU), protocol no. 030/20. For this study, 48 rats (Rattus norvegicus, Wistar strain) aged 3 months, weighing between 250-300 g, were kept in an environment with controlled temperature (21±1ºC), humidity (65-70%), and light cycles (12 hours). The animals were fed appropriate food and had access to water *ad libitum*. This study was conducted according to the ARRIVE guidelines for conducting preclinical studies.

### Groups

The animals were randomly divided into four groups, with 12 animals each, evaluated at two experimental moments (30 and 90 days - n=6). The groups were categorized according to the PBMT protocol used to irradiate the grafted area: CTR: no adjunctive treatment; IRL: PBMT with an infrared laser; RL: PBMT with red light; IRL/RL: PBMT with both infrared and red lasers.

### Surgical procedure

After one week of acclimation to the vivarium environment, the animals were anesthetized with a combination of Ketamine and Xylazine, at a ratio of 80 mg/kg body weight of Ketamine Hydrochloride (Ketamine Hydrochloride 50 mg/ml) and 10 mg/kg body weight of Xylazine Hydrochloride (Xylazine Hydrochloride 20 mg/ml), respectively. Subsequently, the animals underwent trichotomy in the masseteric and submandibular regions, and antisepsis of the surgical field was performed with sterile gauze soaked in povidone solution, with the animal positioned in dorsal decubitus on the surgical table.

Horizontal incisions were made in the lower region of the mandibular ramus, and the muscle tissue and periosteum were elevated to expose the lateral portion of the ascending ramus of the mandible. Four perforations, 0.5 mm in diameter, were made using a spherical bur. These perforations were made parallel to the mandibular base and spaced 6 mm apart, forming the edges of a square. A customized Teflon capsule, shaped like a dome, with an external diameter of 5 mm, a height of 2.5 mm, and a peripheral collar of 1 mm, was inserted with its open portion facing the lateral face of the mandible (two capsules per animal, one on the right side and one on the left side). A volume of 0.032 mm³ of deproteinized bovine bone (Cerabone, Botiss, Zossen, Germany) was compacted inside the capsules, which were then secured to the mandible with 4-0 silk sutures that passed through the capsule and the perforations made in the mandibular ramus. The soft tissues were repositioned over the capsule and sutured with 4-0 Vicryl thread. Postoperatively, the animals received an intramuscular dose of a pentabiotic (0.8 ml/kg) and three doses of 1% ketoprofen (5 mg/kg) administered every 24 hours. The animals were euthanized through an overdose of anesthesia at 30 or 90 days after the surgeries, and the capsule of the right side was used for microtomography analysis, while the capsule of the left side was used for histological analysis.

### Individual PBMT with infrared (808nm) and red laser (660nm)

The GaAlAs laser (Therapy EC, λ 660/808 nm, 100 mW, ϕ ∼0.600 µm, tip divergence = 0.37 rad, CW, spot area of 0.0283 cm², DMC Equipamentos, São Carlos, SP, Brazil) was used for the PBMT application in the groups RL and IRL. In the area that received the graft, four irradiations were performed at equidistant points 3 mm apart, covering the entire grafted area after tissue suturing in a single session for 10 seconds per point, totaling 40 seconds of irradiation. The energy applied at each point was 1 J, resulting in a total of 4 J per session. The energy density used for irradiation was approximately 35.33 J/cm² per point, for a total of 141.32 J/cm².

### Associated PBMT with infrared (808nm) and red lasers (660nm)

The GaAlAs laser (Therapy EC, λ 660 nm/808 nm, 100 mW, ϕ ∼0.600 µm, tip divergence = 0.37 rad, CW, spot area of 0.0283 cm², DMC Equipamentos, São Carlos, SP, Brazil) was used for the PBMT application in the group IRL/RL. In the area that received the graft, four irradiations were performed at equidistant points, 3 mm apart, covering the entire grafted area after tissue suturing in a single session, for 5 seconds at each point, totaling 20 seconds of irradiation. The energy applied at each point was 1 J, resulting in a total of 4 J per session. The energy density used for irradiation was approximately 35.33 J/cm² per point, for a total of 141.32 J/cm².

### Microtomography (µCT)

After the 30 and 90-day periods, the animals were euthanized through intraperitoneal injection of 150 mg/kg of Thiopental combined with 10 mg/kg of Lidocaine. The mandibular ramus samples were fixed in 4% paraformaldehyde for 48 hours and subsequently stored in 70% Alcohol. The samples were then scanned using a Skyscan device (SkyScan, Kontich, Belgium) with the following parameters: Camera pixel: 12.45; X-ray tube power: 65 kVp; X-ray intensity: 385 µA; integration time: 300 ms; filter: Al-1 mm; and voxel size: 18 µm³. The generated images were later reconstructed, spatially reoriented, and analyzed using specific software (NRecon/DataViewer/CTan, Skyscan, Aartselaar, Belgium). The region of interest (ROI) encompassed all the tissue between the dome and the lateral face of the mandibular ramus. A threshold range of 65-250 gray levels was used to assess the volume of mineralized tissue (%BV/TV), number of trabeculae (Tb.N), trabecular thickness (Tb.Th), and space between trabeculae (Tb.Sp) within the ROI. The samples were evaluated by a blinded, experienced, and trained evaluator (GJO).

### Histological Description and Histomorphometry

After µCT analysis, the samples were decalcified in 7% EDTA and processed for paraffin embedding. The samples were sectioned at their central region and embedded in the transverse plane. Serial sections of 5 µm thickness were cut, yielding five histological slides with three sections each, which were stained with hematoxylin-eosin (HE). Three equidistant sections (20 µm apart) were selected, with the first section chosen randomly. Histological images were scanned at 200x magnification using a slide scanner (Aperio Scanscope AT, Leica Biosystems, Germany) and subsequently analyzed using image analysis software (Aperio ImageScope, Leica Biosystems, Germany). The percentages of bone, biomaterial, and soft tissue in the space between the dome and the lateral face of the mandibular ramus were determined. The samples were evaluated by a blinded, experienced, and trained evaluator (GJO).

A histological description was performed to assess the bone and connective tissue components and their relationship to the bone substitute particles. The profile of the infiltrating inflammatory process was also described. This evaluation was performed by an experienced, trained evaluator using magnifications of 25x, 50x, and 200x (GJO).

### Statistical analysis

The numerical data of this study were subjected to the Shapiro-Wilk normality test. The data from the analyses were normally distributed; therefore, a parametric two-way ANOVA, complemented by the Tukey test, was applied. Jamovi v2.3.28 software was used for statistical analysis. All tests were performed with a 95% confidence level.

## Results

**Figure. 1:**
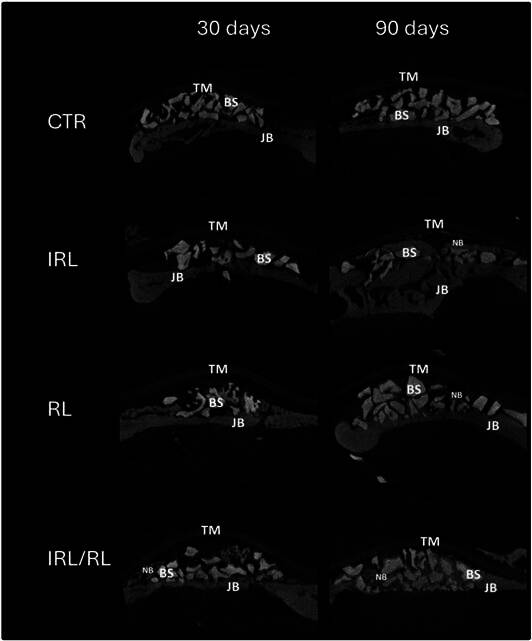
Representative images of the microtomographic analysis in all groups and evaluation moments. JB - Jaw Bone; BS - Bone substitutes; NB - New bone; TM - Teflon Membrane

The IRL/RL group presented greater trabecular thickness than the CTR group after 90 days (p<0.05), while the CTR group had a higher number of trabeculae than the IRL group after 90 days (p<0.05). An increase in BV/TV and Tb.Th was observed in all PBMT-treated groups after 90 days compared to 30 days (p<0.05). Additionally, the CTR group showed an increase in Tb.N and a reduction in Tb.Sp after 90 days compared to 30 days (p<0.05) (Figure 1). Table 1 presents the mean and standard deviation values of bone microstructure data evaluated by microtomographic analysis.

**Table 1:** Mean and standard deviation of bone microstructure data evaluated by microtomographic analysis. *p<0.05 - Differences at 90 days compared to 30 days; Different letters represent different levels of statistically significant differences between groups at each evaluation moment - Two-way ANOVA complemented by the Tukey test. Volume of mineralized tissue (%BV/TV), number of trabeculae (Tb.N), trabecular thickness (Tb.Th), and space between trabeculae (Tb.Sp).

Moment	Groups	BV/TV	Tb.Th	Tb.N	Tb.Sp
30d	CTR	52.9±8.0	14.0±2.6	0.04±0.002	15.5±2.6
	IRL	41.0±4.4	13.3±0.8	0.03±0.001	17.3±3.0
	RL	37.4±11.7	11.7±0.9	0.03±0.01	21.1±6.9
	IRL/RL	43.4±12.0	12.7±1.0	0.03±0.008	13.9±1.0
90d	CTR	47.6±10.5	14.1±0.6^b^	0.04±0.004^*a^	12.0±2.3^*^
	IRL	51.0±7.0^*^	15.7±0.5^*ab^	0.03±0.004^b^	16.4±3.1
	RL	51.2±7.6^*^	14.5±1.0^*b^	0.03±0.007^ab^	15.0±3.7
	IRL/RL	60.2±9.6^*^	16.1±1.3^*a^	0.03±0.003^ab^	12.6±4.04

**Figure 2:**
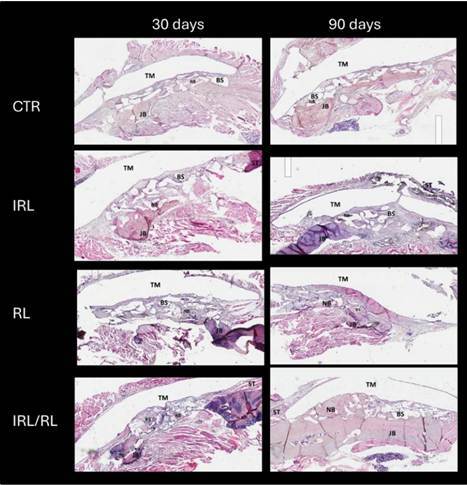
Representative histological images in all groups and evaluation moments. JB - Jaw Bone; ST - Soft tissue; BS - Bone substitutes; NB - New bone; TM - Teflon Membrane. HE (25x - Original magnification)

Regarding histomorphometric analysis, the IRL/RL group showed greater bone volume after 90 days than all other groups (p<0.05) and less soft tissue than the CTR and RL groups after 90 days (p<0.05) (Figure 2). Table 2 presents the mean and standard deviation values of the composition data for the grafted areas evaluated through histomorphometric analysis.

**Table 2:** Mean and standard deviation of the composition data for the grafted areas evaluated through histomorphometric analysis. Different letters indicate statistically significant differences between groups at each evaluation moment - Two-way ANOVA complemented by the Tukey test. Volume of mineralized tissue (%BV/TV), number of trabeculae (Tb.N), trabecular thickness (Tb.Th), and space between trabeculae (Tb.Sp).

Moment	Groups	%Bone	% Bone substitute	%Soft tissue
30d	CTR	20.0±4.4	29.1±4.9	50.9±6.5
	IRL	26.6±5.4	29.4±5.0	44.0±5.5
	RL	21.7±5.9	29.5±9.2	48.7±9.7
	IRL/RL	28.3±7.4	28.3±6.7	43.4±4.9
90d	CTR	20.8±4.2^b^	31.6±4.7	47.6±3.2^b^
	IRL	25.4±3.0^b^	30.8±6.6	43.8±6.7^ab^
	RL	23.1±3.7^b^	27.3±4.6	49.5±5.5^b^
	IRL/RL	32.0±4.9^a^	30.1±2.9	37.9±6.6^a^

Regarding histological findings, at 30 days, the grafted area generally showed low bone formation, located close to the native bone. In all groups, particles near the native bone were in contact with the new bone; however, in the area near the Teflon membrane, the bone substitute particles were embedded in the connective tissue matrix. In the RL groups, a moderate inflammatory infiltrate and a less organized connective tissue matrix were observed in some sections. In contrast, the other groups showed a lower rate of inflammatory infiltrate.

**Figure 3:**
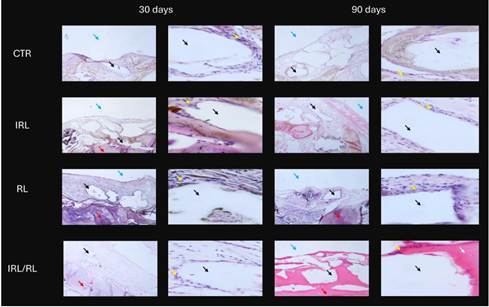
Representative histological images in all groups and evaluation moments. Red Arrow - Jaw Bone; Yellow Arrow - Connective tissue matrix; Black Arrow - Bone substitutes; White Arrow - New bone; Blue Arrow - Teflon Membrane. HE (50x and 200x magnification).

It was observed at 90 days that an increase in the organization of the connective tissue matrix around the bone substitute particles was located further from the native bone. Specifically, in the IRL-RL group, bone formation was observed around the particles in the aforementioned regions. In the area close to the native bone, the new mineralized bone was observed in the graft areas in all the groups (Figure 3). The connective tissue was well-organized in all groups; however, a denser fiber pattern was observed around the bone substitute particles in the IRL and CTR groups, whereas in the RL group, the connective tissue was more immature. A low rate of inflammatory infiltrate was observed in all the groups (Figure 3).

## Discussion

In this study, microtomography analysis showed an increase in BV/TV in all PBMT-treated groups after 90 days compared to 30 days (p<0.05). Furthermore, histomorphometry analysis showed that the IRL/RL group had greater bone volume after 90 days than all other groups (p<0.05). PBMT with low-intensity lasers has shown promising results in animal models as an adjunct therapy to accelerate bone repair in sites grafted with deproteinized bovine bone ^16^, ^11^, ^13^. In the current study, it was observed that the application of PBMT with dual wavelength promoted greater bone tissue formation in sites grafted with deproteinized bovine bone compared to the other groups. Additionally, a reduction in the proportion of soft tissues and greater trabecular thickness were observed, indicating higher-quality grafted tissue with greater potential to facilitate, for example, osseointegration. This effect of combining red and infrared laser PBMT may be due to the combined effect of these different wavelengths on the different tissues targeted ^11^, ^13^. More studies using this methodology are needed to determine whether additional irradiation sessions would further improve the assessed parameters; however, it is worth considering the patient's adherence to treatment, as more follow-ups would be required.

The superior outcomes in the grafted areas' healing promoted by the dual-wavelength PBMT may be due to the effects of the red and infrared lasers in different regions of the surgical site. Although protein and gene expression analyses were not performed in this study, the biological events induced by PBMT have been previously reported. The mechanisms of action of these different wavelengths are similar ^25^ and involve upregulation of bone and soft tissue growth factors (e.g., TGFβ1; BMP2) and downregulation of proinflammatory mediators (e.g., TNFα; IL1β) ^11^, ^22^. However, the depth of the effect differs: the red laser promotes a greater effect on superficial tissues, while the infrared laser induces its effects in deeper tissues ^23^, ^24^. A recent study found that PBMT accelerated soft-tissue repair in patients who had undergone gingivectomy ^17^. This effect may have facilitated healing of the soft tissues covering the grafted area, thereby aiding bone tissue formation, as inadequate soft tissue healing impairs bone tissue formation ^18^. In turn, PBMT with an infrared laser has previously demonstrated effects on the healing of grafted and ungrafted areas and on the acceleration of osseointegration in both native and grafted bone areas ^19^. Another study showed that PBMT with an infrared laser improved bone repair in sites grafted with xenogenic and synthetic bone substitutes ^11^. Therefore, the effect observed in the current study may have resulted from the combined effects of these two wavelengths.

Intriguingly, a previous preclinical study showed that PBMT with an infrared laser increased bone tissue formation in areas grafted with DBB, using a model similar to that used in the current study ^11^. The discrepancy in the outcomes may have occurred for two reasons: 1) The number of sessions of PBMT was lower in the present study; 2) the deproteinized bovine bone (DBB) used in this study is manufactured at high temperatures (1,200°C), whereas the DBB used in the previous study is sintered at 400°C. The differing crystallinity of these materials affects their osteoconductive properties. Thus, the lower degree of osteoconduction observed in the DBB in this study may account for the lack of observed effects of PBMT with infrared light on bone repair.

One central question regarding the results is the fact that differences between groups were only found at the 90-day evaluation, which can be explained by the challenging nature of the defect for the repair process, necessitating a more extended period to notice differences in bone tissue formation stimulated by PBMT, as the laser accelerates cellular metabolism by acting directly on mitochondria and regulates bone metabolism through the expression of regulatory proteins ^20^. These effects do not occur immediately, which justifies why the effects of PBMT were observed over more extended periods ^11^.

The current study has limitations that should be considered when evaluating the findings. Several studies have already demonstrated the efficacy of PBMT with low-intensity lasers as an adjunct therapy in guided bone regeneration procedures ^16^, ^11^, ^12^. However, no studies have compared the laser penetration capacity through the different membranes and barriers used in this procedure, and this should be further studied. Additionally, it was noted that microtomographic analysis has limitations in precisely distinguishing mineralized tissue from biomaterial particles, which may have affected the visualization of bone tissue structure and quantity ^21^. These results were influenced by the presence and structure of the bone substitute rather than by the newly formed tissue, potentially hindering the visualization of differences in bone microstructure among the groups evaluated in this study.

PBMT with dual-wavelength (red and infrared) irradiation in a single intraoperative session improved bone repair at sites grafted with deproteinized bovine bone in the mandibles of rats.
